# Multiple Myeloma: Available Therapies and Causes of Drug Resistance

**DOI:** 10.3390/cancers12020407

**Published:** 2020-02-10

**Authors:** Vanessa Pinto, Rui Bergantim, Hugo R. Caires, Hugo Seca, José E. Guimarães, M. Helena Vasconcelos

**Affiliations:** 1 i3S–Instituto de Investigação e Inovação em Saúde, Universidade do Porto, 4200-135 Porto, Portugal; vanessa_pinto_92@hotmail.com (V.P.); rbergantim@ipatimup.pt (R.B.); hcaires@ipatimup.pt (H.R.C.); h_seca@hotmail.com (H.S.); jeguimaraes@hsjoao.min-saude.pt (J.E.G.); 2 Cancer Drug Resistance Group, IPATIMUP–Institute of Molecular Pathology and Immunology of the University of Porto, 4200-135 Porto, Portugal; 3 FCTUC–Faculty of Science and Technology of the University of Coimbra, 3030-790 Coimbra, Portugal; 4 Clinical Hematology, Hospital São João, 4200-319 Porto, Portugal; 5 Clinical Hematology, Faculty of Medicine, University of Porto, 4200-319 Porto, Portugal; 6 Department of Biological Sciences, FFUP-Faculty of Pharmacy, University of Porto, 4050-313 Porto, Portugal

**Keywords:** multiple myeloma, drug resistance, drug response, treatment

## Abstract

Multiple myeloma (MM) is the second most common blood cancer. Treatments for MM include corticosteroids, alkylating agents, anthracyclines, proteasome inhibitors, immunomodulatory drugs, histone deacetylase inhibitors and monoclonal antibodies. Survival outcomes have improved substantially due to the introduction of many of these drugs allied with their rational use. Nonetheless, MM patients successively relapse after one or more treatment regimens or become refractory, mostly due to drug resistance. This review focuses on the main drugs used in MM treatment and on causes of drug resistance, including cytogenetic, genetic and epigenetic alterations, abnormal drug transport and metabolism, dysregulation of apoptosis, autophagy activation and other intracellular signaling pathways, the presence of cancer stem cells, and the tumor microenvironment. Furthermore, we highlight the areas that need to be further clarified in an attempt to identify novel therapeutic targets to counteract drug resistance in MM patients.

## 1. Introduction

Multiple myeloma (MM) is a rare blood disease, representing 1% of cancers and 10% of all hematological malignancies, being the second most common blood cancer [1,2]. MM is frequently associated with the elderly, as the majority of the patients are diagnosed between the age of 60 and 70; however, in recent years younger patients have been also diagnosed [3,4]. The median overall survival increased from 2–3 years to 8–10 years [5,6], mainly due to the better understanding of the disease biology and its heterogeneity [7], the use of autologous stem cell transplant, introduction of novel therapeutic drugs and a better use of them [5,6,8].

MM is characterized by bone marrow (BM) infiltration of monoclonal plasma cells (PC), which secrete monoclonal immunoglobulin (Ig) that can be found in the blood and/or urine. The accumulation of these immunoglobulins will lead to organ dysfunction, usually referred to as C-R-A-B (hypercalcemia, renal insufficiency, anemia and bone lesions) [2,9,10] and at this stage patients will become symptomatic. 

Normally the PCs, representing the final stage of maturation of B-cells, produce polyclonal immunoglobulins to fight infections [11]. The maturation of B lymphocytes occurs in the BM and afterwards migrate to secondary lymph nodes, where antigens are presented to B cells. Immature PCs are characteristically short-lived cells and producers of IgM involved in the primary immune response [11,12]. In some circumstances, PC experience hypermutations of the Ig light (IgL) and heavy chains (IgH) variable genes, secreting other Ig isotypes, such as IgG and IgA, or infrequently IgE and IgD. Later, these cells migrate to the BM to differentiate into long-lived PCs, lasting for days or months.

**Figure 1:**
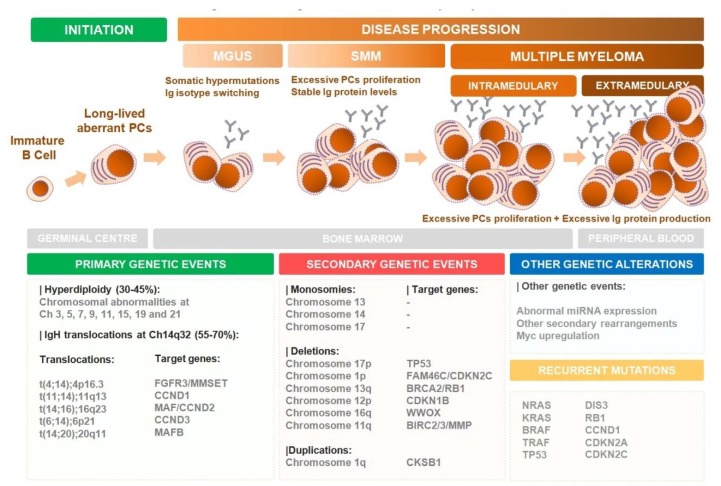
Biology of multiple myeloma (MM) development and progression. MM is the end stage of a multistep neoplastic transformation of PCs. Virtually all MM initiates as a monoclonal gammopathy of undetermined significance (MGUS). In this early stage, a BM PC may accumulate several primary genetic mutations (as chromosomal abnormalities and/or IgH translocations) that affect the expression of key target genes (e.g., cyclins, FGFR3, MYC deregulation, etc.) granting a proliferative advantage to these mutated cells. The presence of abnormal amounts of PCs in the BM with slightly increased levels of Ig proteins but no clinical symptoms is defined as smoldering multiple myeloma (SMM). Nevertheless, these hyperproliferative PCs will endure additional secondary genetic mutations that aggravate this aberrant phenotype leading to the accumulation of high amounts of PCs in the BM and consequently to the secretion of excessive levels of Igs towards the blood stream. Ultimately, this will lead to the clinical manifestation of severe symptoms (as hypercalcemia, renal insufficiency, anemia and bone lesions) that define MM. This disease may progress to extramedullary disease in more advanced stages. Additional aberrant genetic events, such as mutations, deletions, methylations and microRNA (miRNA) abnormalities may occur during MM development defining the aggressiveness of the disease and response to therapy.

When monoclonal PCs abnormally proliferate and overproduce large amounts of immunoglobulins, the MM occurs [10,12,13] (Figure 1). Hypermutations and atypical isotype switching are potential initial genetic events for MM development and prone the appearance of major translocations and other chromosomal anomalies, mainly trisomies. 

From a genetic perspective, a first separation can be made between: (i) hyperdiploid karyotype, related to numerous chromosomal abnormalities (chromosomes 3, 5, 7, 9, 11, 15, 19 and 21) but rare IgH translocations; and (ii) non-hyperdiploid karyotypes, well-defined by the occurrence of IgH translocations [14,15,16]. Additional aberrant genetic events, such as mutations, deletions, methylations and microRNA (miRNA) abnormalities, usually occur later in the MM development [17,18,19,20].

MM ontogeny is characterized by different stages of disease. The first stage, called monoclonal gammopathy of undetermined significance (MGUS), presents a low rate of plasma cell (PC) proliferation, low immunoglobulin burden and no clear related symptoms [21,22]. Patients with MGUS may develop MM, at a rate of 1% per year at 20 years [23]. In some patients, it is also possible to identify an intermediate stage classified as smoldering multiple myeloma (SMM), with higher immunoglobulin (Ig) burden but likewise asymptomatic [24]. When patients become symptomatic, the disease is referred to as MM, which may be either intramedullary or extramedullary [1,2]. The extramedullary disease is associated with a worse prognosis, mainly the widespread stage called plasma cell leukemia, in which we can find high levels of malignant PCs circulating in the peripheral blood [25,26].

## 2. Treatment of Multiple Myeloma

**Figure 2:**
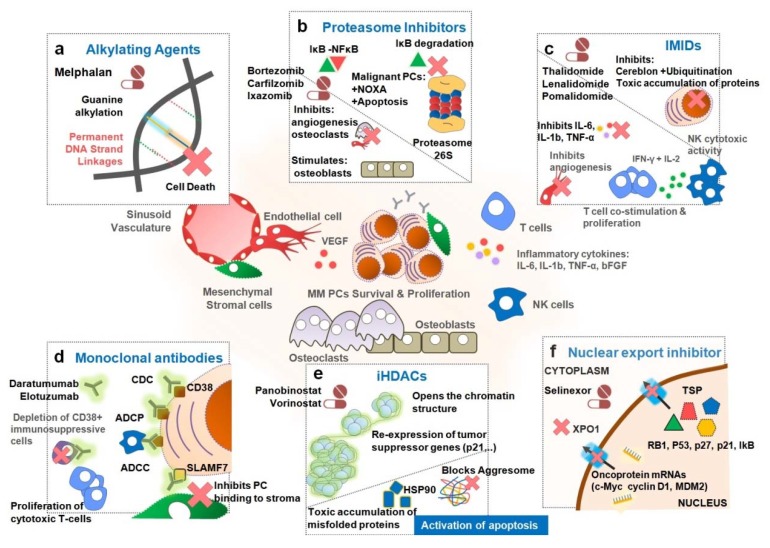
Therapeutic options to counteract MM. Treatments used for symptomatic or active MM include corticosteroids, alkylating agents, proteasome inhibitors (PIs), immunomodulatory drugs (IMIDs), monoclonal antibodies (mAbs), histone deacetylase inhibitors (iHDACs) and nuclear export inhibitors. (**a**) Classical alkylating agents as melphalan target highly proliferating cells, including malignant PCs, by intercalating permanently their DNA, causing cell death later on. (**b**) PIs as bortezomib, carfilzomib and ixazomib block the IkB and/or pro-apoptotic proteins degradation in malignant plasma cells proteosome, overcoming their resistance to apoptotic stimuli. (**c**) IMIDs as thalidomide, lenalidomide and pomalidomide modulate the inflammatory environment of the BM inhibiting the progression of MM [e.g., reduction of Interleukin-6 (IL-6), tumor necrosis factor-α (TNF-α), etc.] through inhibition of angiogenesis and other key stromal-MM cell interactions). Some of these drugs target the cereblon protein of the E3 ubiquitin ligase complex blocking the ubiquitination process in malignant PCs. This in turn leads to a toxic accumulation of proteins and cell death. (**d**) Monoclonal antibodies (mAbs) as daratumumab, isatuximab and elotuzumab bind to specific antigens on the surface of malignant PCs. This will in turn induce MM plasma cell death by antibody-dependent cell-mediated cytotoxicity (ADCC), complement-dependent cytotoxicity (CDC) and/or antibody-dependent cellular phagocytosis (ADCP). (**e**) histone deacetylase inhibitors (iHDACs) such as panobinostat and vorinostat act on malignant PCs by opening the chromatin structure. Consequently, this will activate the expression of tumor suppressor genes, which were previously silenced by aberrant histone acetylation in malignant PCs. (**f**) Exportin 1 (XP01) inhibitors as selinexor act on malignant PCs by blocking the export tumor suppressor proteins out of the nucleus by the XPO1 pump while retaining many oncoprotein mRNAs within the nucleus.

Treatment of MM usually includes different combinations of drugs having different mechanisms of action—corticosteroids, alkylating agents, anthracyclines, proteasome inhibitors (PIs), immuno-modulatory drugs (IMIDs), histone deacetylase inhibitors (iHDACs), monoclonal antibodies (mAbs), nuclear export inhibitors and high-dose chemotherapy rescued by autologous stem cell transplantation (ASCT) [27,28]. Most of these therapeutic options are represented in Figure 2. For many years, therapies were based on cytotoxic drugs, mainly melphalan, an alkylating agent (Figure 2a), and prednisone, a corticosteroid [29]. Later, high-dose chemotherapy (HDT) with melphalan rescued by ASCT was introduced, resulting in extended overall survival for younger patients when compared with conventional chemotherapy [30]. For elderly patients, the most used treatment consisted of the oral combination of melphalan and prednisone. Nonetheless, over the last two decades, new drugs such as thalidomide, bortezomib and lenalidomide were included in the treatment of these patients [31]. The use and optimization of combinations of these drugs allowed improving the overall survival of MM patients – the median survival was of 2.5 years prior to 1997, 4 years in the following decade and over 7 years more recently [5,8,32]. However, resistance to these agents has been observed and MM patients who became refractory to both first generation IMIDs and PIs have significant worse outcomes [33]. Furthermore, next generation IMIDs and PIs were developed and introduced in the therapy of relapsed and refractory patients, and more recently, regimens combining the use of mAbs and iHDACs were also approved for MM treatment [34,35]. Consequently, the selection of the primary treatment has changed over time. Patients are usually stratified by age, performance status and comorbidities, in order to evaluate potential candidates for ASCT [9,36,37]. Advanced age and frailty of patients are life-threatening factors [38]. Nonetheless, age should not limit the access to new drugs or treatment modalities. Elderly fit patients also benefit from ASCT, as younger patients do [37,39]. Disease aggressiveness, namely cytogenetics and extramedullary manifestations, also guide the choice of the best treatment approach. Patients with high-risk features [del17p, t(14;16) and t(4;14)] have a median overall survival of 2 to 3 years, even with ASCT, while MM patients without these features have better survival prognosis, of 6 to 7 years [40,41]. Therefore, patients with high-risk myeloma receive denser and more intense treatments [42,43].

Currently, newly diagnosed MM (NDMM) patients are treated with induction therapy that usually includes triple combinations, such as bortezomib, lenalidomide and dexamethasone. Other preferred regimens may be selected depending on the eligibility of the candidates for transplants [44,45]. This first phase of treatment has the objective of reducing tumor burden and improving the hematopoietic stem cell collection [46]. Since the melphalan therapy can interfere with adequate stem-cell mobilization, melphalan-based regimens are no longer a standard treatment, neither for younger nor for elder patients, nor for those who are not eligible for ASCT [1]. After achieving response, this primary therapy is followed by high dose melphalan and ASCT, consolidation and/or maintenance using bortezomib or lenalidomide, according to local protocols [9,44,45].

For relapsed MM patients, the situation has also improved with new treatment options being recently approved, new strategies for therapy sequence and novel double/triple combinations [47,48,49,50].

### 2.1. Proteasome Inhibitors (PIs)

#### 2.1.1. Bortezomib

Bortezomib was the first PI approved for the treatment of NDMM patients and for relapsed and refractory MM (Figure 2b) [51,52,53]. The introduction of this PI represented a breakthrough in the treatment of MM, by preventing pro-apoptotic proteins degradation and leading to the apoptosis of malignant cells [52]. Additionally, by blocking the degradation of inhibitor kappa B (IκB), the inhibitor of nuclear factor B (NFκB), bortezomib suppresses the NFκB signaling pathway preventing the activation of numerous anti-apoptotic genes involved in MM progression [52,53]. Bortezomib also leads to the upregulation of NOXA, a pro-apoptotic member of the B-cell lymphoma 2 (Bcl-2) protein family, which interacts with the anti-apoptotic proteins of the same family [B cell lymphoma-extra large (BCl-X_L_) and Bcl-2], inducing the apoptosis of the myeloma cells [52]. Bortezomib also inhibits osteoclasts and stimulates osteoblasts, thereby increasing bone formation [54].

#### 2.1.2. Carfilzomib

Carfilzomib is a next generation PI approved for the treatment of patients who relapsed or became refractory. Carfilzomib binds irreversibly to the proteasome, leading to a potent inhibition of proliferation and induction of apoptosis even in bortezomib-resistant MM cell lines and patient’s samples [55]. The approval of carfilzomib as a single agent was based in studies that showed higher response rates in relapsed or refractory MM patients [56,57]. The combination of carfilzomib with dexamethasone or with lenalidomide plus dexamethasone was also approved after results showing significant improvement of the progression-free survival in patients who had received previous therapies but relapsed [58,59]. In some countries this regimen is also considered in NDMM patients, since it was shown to be effective and well tolerated [60].

#### 2.1.3. Ixazomib

Ixazomib is the first oral PI approved for the treatment of relapsed and refractory MM. As the other PIs, ixazomib promotes caspase-dependent induction of apoptosis and inhibition of cell cycle, inhibits the NF-κB pathway in MM cells and inhibits tumor-associated angiogenic activity [61]. The addition of ixazomib to lenalidomide and dexamethasone significantly increased the progression-free survival of patients treated with this regimen, when compared with those who received lenalidomide plus dexamethasone [62]. In NDMM patients, ixazomib also showed promising results, being effective and apparently well tolerated by these patients [63].

### 2.2. Immunomodulatory Drugs (IMIDs)

#### 2.2.1. Thalidomide

Thalidomide has several effects on the immune system by modulating its components, enhancing immune surveillance and changing the inflammatory BM microenvironment [64]. Benefits of thalidomide in MM are reflected by its ability to disturb the interactions between myeloma cells and the BM microenvironment [65], e.g., by inhibiting interleukin-6 (IL-6), interleukin 1β (IL-1β), basic fibroblast growth factor (bFGF) and vascular endothelial growth factor (VEGF) expression, all of which are essential for the growth of myeloma cells (Figure 2c) [65,66]. Thalidomide, by modulating T cells, also decreases the levels of the tumor necrosis factor-α (TNF-α), another cytokine important for MM cells growth and survival; and by affecting other immune cells, this IMID inhibits *de novo* IgM antibody synthesis [64]. Thalidomide additionally induces T-cell proliferation, by secreting interferon gamma (IFN-γ) and Interleukin-2 (IL-2) [64,66,67]. For many years, the mechanism of action and targets of thalidomide and its derivatives, such as lenalidomide (see 2.2.2.) was completely unknown. Recently, it was found that IMIDs bind to a primary protein target termed cereblon, which belongs to an E3 ubiquitin ligase complex. Therefore, the thalidomide inhibition of the ubiquitination process leads to the toxic accumulation of proteins and to MM cell death [68]. Novel findings associate cereblon with other downstream targets, participating in the binding, ubiquitination and degradation of Ikaros (IKZF1) and Aiolos (IKZF3), two transcription factors that maintain MM cells function [69,70,71]. Accordingly, MM cells lacking cereblon become highly resistant to IMIDs [72].

#### 2.2.2. Lenalidomide

Lenalidomide is more potent and effective than thalidomide in modulating the immune system [64]. The secretion of cytokines increases MM growth and survival, being associated with drug resistance [64,66]. Lenalidomide inhibits the production of pro-inflammatory cytokines such as IL-6, TNF-α, Interleukin-1 (IL-1) or Interleukin-12 (IL-12), and promotes the production of the anti-inflammatory cytokine IL-10 [64]. Like thalidomide, it inhibits the adhesion of MM to bone marrow stromal cells (BMSCs) and, consequently, decreases the production of IL-6 and downregulates TNF-α production (decreasing its levels up to 50,000 times more than thalidomide [64,65]. As thalidomide, it co-stimulates about 50 to 2000 times more T-cell proliferation triggered by the T cell receptor, increasing by 50 to 100 times the secretion of IFN-γ and IL-2 [64,65]. Besides the clonal production of both cytotoxic CD8+ and helper CD4+ T cells, lenalidomide also enhances natural killer (NK) cell activity against MM cells [64,65,73]. Lenalidomide blocks angiogenesis (being 2 to 3 times more potent than thalidomide as an antiangiogenic drug) by decreasing the angiogenic factors VEGF and IL-6 [64], and consequently inhibiting the development of blood vessels required for the growth of primary and metastatic tumors [65].

#### 2.2.3. Pomalidomide

Like others IMIDs, pomalidomide acts by inhibiting MM cells proliferation and by inducing apoptosis. Likewise lenalidomide, it also enhances T-cell and NK cells activity, inhibits the production of pro-inflammatory cytokines and demonstrates anti-angiogenic activity, being also more potent than thalidomide. In order to produce its effects, it also requires the presence of cereblon in the MM cells [70,71,72,73,74]. Pomalidomide efficacy is higher when combined with dexamethasone or with PI combinations such as bortezomib. Nowadays, pomalidomide should be considered a beneficial treatment option for relapsed and refractory MM patients who received prior therapies that included bortezomib or lenalidomide [75,76,77].

### 2.3. Monoclonal Antibodies (mAbs)

#### 2.3.1. Anti-CD38

Monoclonal antibodies bind to specific antigens on the surface of cells, inducing tumor cell death by antibody-dependent cell-mediated cytotoxicity (ADCC), complement-dependent cytotoxicity (CDC) and antibody-dependent cellular phagocytosis (ADCP). The majority of mAbs are associated with cell death mediated by Fc gamma receptor (FCyR) crosslinking of tumor-bound antibodies and modulation of target antigen enzymatic activity (Figure 2d) [78,79]. Daratumumab, isatuximab and elotuzumab were the first mAbs introduced in the clinic for the treatment of MM [80].

Daratumumab targets the cell surface marker CD38, which is highly expressed on MM cells, and induces cellular cytotoxicity through different immune-mediated mechanisms leading to the lysis of those CD38-positive MM cells [79,81]. Patient’s response to daratumumab is influenced by CD38 expression levels with reduced CD38 levels conferring resistance [79]. Daratumumab also reduces the immunosuppressive activity of regulatory T and B cells, with an increase in the number of cytotoxic T-cells being observed in relapsed and refractory patients [79].

The efficacy, safety and clinical activity of daratumumab as monotherapy was demonstrated in relapsed and refractory MM patients previously submitted to two or more therapies with PIs and IMIDs [82,83]. These studies supported the single agent daratumumab approval in 2015 [82,83], by providing very promising results for relapsed or refractory patients who had been heavily pretreated and had particularly poor outcomes [33,84]. In relapsed or refractory patients, daratumumab was also approved for triple combinations, either with lenalidomide and dexamethasone [85], bortezomib and dexamethasone [86] or pomalidomide plus dexamethasone [87], showing an important beneficial effect when compared with the same regimens without daratumumab. More recently, several randomized trials showed a significant benefit for NDMM patients when adding daratumumab to the standard regimens with bortezomib, melphalan and prednisone [88], bortezomib, thalidomide and dexamethasone [89] or lenalidomide and dexamethasone [90].

Isatuximab binds selectively to CD38, thus promoting MM cell death [91]. Unlike daratumumab, it induces apoptosis independently from cross-linking agents [92]. In the clinical practice, isatuximab has showed promising data when combined with pomalidomide in refractory and relapsed patients [93], and also with bortezomib and lenalidomide in first line treatment [94]. 

#### 2.3.2. Elotuzumab

Elotuzumab targets the CS1, a glycoprotein present in the surface of MM cells, also named signaling lymphocytic activation molecule family member 7 (SLAMF7). Elotuzumab targets MM cells via ADCC and, through the action of NK cells, promotes tumor cells death and reduces the MM cell binding to the bone marrow stroma [95,96]. Elotuzumab was evaluated in a monotherapy study but showed no activity as a single agent in MM patients [97]. However, when combined with lenalidomide and low-dose dexamethasone, elotuzumab demonstrated clinical efficiency, which then supported the approval of this regimen for the treatment of patients with relapsed or refractory MM [98,99]. 

### 2.4. Histone Deacetylase Inhibitors (iHDACs)

#### 2.4.1. Panobinostat

Panobinostat is a potentiHDAC, promoting the opening of chromatin structure and consequently activating the expression of tumor suppressor genes, which had previously been silenced by aberrant histone acetylation (Figure 2e) [100]. Preclinical research with proteasome and iHDAC showed synergistic activity with proteasomal and aggresomal protein degradation systems, resulting in accumulation of polyubiquitinated proteins and activation of apoptosis [101]. Other mechanisms such as the upregulation of factors that block cell cycle promotion (p21) and regulation of proapoptotic and antiapoptotic proteins or even of caspase mediated-direct toxicity, appear to be involved in iHDAC treatment of MM [102]. Panobinostat, in combination with bortezomib and dexamethasone, is already approved for the treatment of patients with relapsed and refractory MM that received at least 2 regimens including PIs and IMIDs [103,104,105].

#### 2.4.2. Vorinostat

Another well studied iHDAC is vorinostat, which has been tested for the treatment of relapsed or refractory MM patients in combination with other agents. So far, the most encouraging results were obtained when combining vorinostat with bortezomib and lenalidomide [106,107,108].

### 2.5. Other Drugs

#### Selinexor

Exportin 1 (XPO1) is a karyopherin responsible for the nuclear export of innumerous cargo proteins, including nearly all tumor suppressor proteins and several oncoproteins (Figure 2f) [109]. In MM, XPO1 is overexpressed resulting in enhanced transport of tumor suppressor proteins out of the nucleus and allowing immune surveillance evasion by cancer cells and escape from cell-cycle regulation [109,110]. Moreover, when XPO1 is complexed with the mRNA cap-binding protein eukaryotic translation initiation factor 4E (eIF4E), transports many oncoprotein mRNAs [such as c-Myc, cyclin D1, murine double minute 2 (MDM2)] to the cytoplasm, leading to their downstream effects in cancer promotion [110,111]. Selinexor is the first-in-class selective inhibitor of XPO1, forcing the nuclear retention and activation of tumor suppressor proteins, trapping IκBα in the nucleus to suppress NF-κB activity, and reducing oncoprotein mRNAs translation. Ultimately, it causes a selective induction of apoptosis in malignant cells, sparing normal cells. Thus, selinexor appears as a promising treatment for MM patient’s refractory to all of the previously mentioned classes of drugs [109,110,112].

## 3. Causes of Drug Resistance in MM Patients

MM patients’ histories are characterized by multiple relapses after different lines of treatment until becoming refractory [113,114]. The main cause for the relapses is drug resistance, which is dramatically associated with an unfavorable prognosis. 

**Figure 3:**
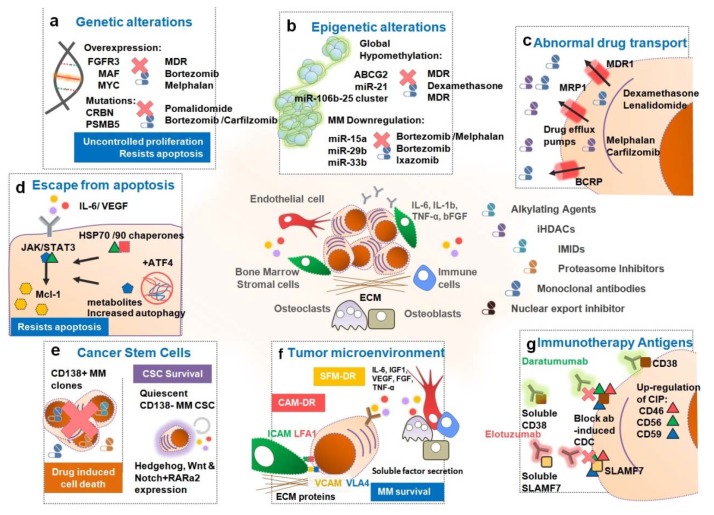
Mechanisms of Drug Resistance enforced by MM. (**a**) Genetic alterations such as t(4;14), t(14;16) and t(14;20) translocations, 17p and 13p deletions and c-Myc associated abnormalities are associated with an unfavorable prognosis and insufficient response to current treatments. Additionally, (**b**) epigenetic alterations induced by a global hypomethylation of the DNA leads to the abnormal expression of several genes such as ATP binding cassette super-family G member 2 (ABCG2) and several miRs (e.g., miR-21, -15a, -29b, etc.) in malignant plasma cells (PCs) conferring a multidrug resistance (MDR) phenotype. (**c**) The overexpression of drug efflux pumps, namely P-glycoprotein (P-gp), in the malignant PCs mediates the cellular efflux of several drugs lowering intracellular drug concentration to sub-lethal levels. Moreover, (**d**) alterations in NF-κB, phosphatidylinositol 3-kinase/ protein kinase B (PI3K/AKT), mitogen-activated protein kinase/ extracellular signal-regulated kinase (MAPK/ERK) and Janus kinase/signal transducer and activator of transcription 3 (JAK/STAT3) signaling pathways in these PCs confers resistance to apoptotic stimuli and evasion to drug-induced cell death. (**e**) CD138- MM putative cancer stem cells are intrinsically resistant to most drugs. This small subset of cancer stem cells (CSCs) will survive therapy and remain as undetected/quiescent residual disease. Later on, these CSCs have the ability to self-initiate MM and cause refractory post-treatment relapse. (**f**) The bone marrow microenvironment is essential for MM survival, development and drug resistance by secretion of soluble factors e.g., interleukin 6 (IL-6), insulin-like growth factor 1 (IGF-1), vascular endothelial growth factor (VEGF), B-cell activating factor (BAFF), fibroblast growth factor (FGF), stromal cell-derived factor 1α (SDF1α), and tumor necrosis factor-α (TNF-α). (**g**) Immunotherapy antigens: the anti-CD38 and SLAM7 monoclonal antibodies (mAbs), daratumumab and elotuzumab, fail to reach therapeutic efficacy either due to extracellular binding of the mAbs to target antigens or to upregulation of cell surface expression of the complement-inhibitors proteins CD46, CD56 and CD59.

There are various causes for drug resistance in MM (Figure 3): (1) genetic alteration; (2) epigenetic alterations [20,115,116]; (3) abnormal drug transport and metabolism, decreasing the intracellular drugs levels [117]; (4) dysregulation of apoptosis or other intracellular signaling pathways and activation of autophagy [114,115]; (5) persistence of cancer stem cells, which are insensitive to most drugs and capable of self-initiating MM [115,118]; (6) dysfunctional tumor microenvironment, enlightened by the dependence of MM cells on the stromal microenvironment components [115,119]; and (7) other specific mechanisms for immunotherapies with antibodies.

Drug resistance in cancer may result from intrinsic mechanisms, in which malignant cells are resistant to therapies even before treatment, or acquired during treatment reflecting the “selective pressure” induced by treatment [120,121]. To date, the reason why patients relapse and how drug resistant MM clones alter their dominance and persist after therapies are not fully understood [120].

Due to the heterogeneous nature of MM, many causes are reported to be involved in MM drug resistance (Table 1). 

### 3.1. Genetic Alterations Influencing Drug Resistance in MM

The genetic, cytogenetic and epigenetic changes related to the MM pathogenesis are associated with predisposition to drug resistance and, eventually, relapse [116,160,161].

The IgH translocations involving the chromosome 14q32 are believed to be one of the first events involved in the MM pathogenesis [162]. Most common partner chromosomes of this translocation are: 4p16.3, resulting in upregulation of fibroblast growth factor receptor 3 (FGFR-3) and multiple myeloma SET domain (MMSET) genes; 11q13, dysregulating cyclin D1 gene (CCND1); 16q23, upregulating the transcription factor musculoaponeurotic fibrosarcoma (MAF) and, consequently the cyclin D2 gene (CCND2); 6p21, upregulating the cyclin D3 gene (CCND3); and 20q11, mediating the transcription factor musculoaponeurotic fibrosarcoma B (MAFB) levels [162,163,164,165,166]. All of these translocations juxtapose IgH gene enhancers next to oncogenes [167]. The resulting unbalanced expression of the mentioned genes will contribute to the malignant phenotype of MM (Figure 3a) [165].

The t(4;14) translocation, occurring in 15% of patients, is associated with therapeutic failure and consequently high rates of relapse [155,168,169]. Treatment failures are due the overexpression of the highly active oncogenes FGFR3 and MMSET [170,171]. FGFR3 is overexpressed in 74% of patients with t(4;14) translocations, while MMSET is overexpressed in all patients [121]. Increased expression of FGFR3 contributes to tumor establishment [172], while the MMSET gene intensifies cell proliferation by decreasing cell cycle arrest, apoptosis and enhancing cell adhesion [171]. 

The t(14;16) and t(14;20) translocations, less frequent among MM patients (in 5% and 2% of cases, respectively) are also associated with unfavorable prognosis [173]. MAF (also named c-MAF) and MAFB are the key oncogenes upregulated in MM cells with t(14;16) and t(14;20) [170,174,175] translocations, respectively. MAF upregulates CCND2 genes, targeting PCs to be more responsive to external stimuli and adhesion signaling to the stroma [176]. Patients with MAF overexpression showed poorer response to bortezomib and carfilzomib. MM cell lines with MAF silencing were identified as more sensitive to proteasome inhibitors and prone to induce apoptosis trough caspase activation, while cell lines with MAF overexpression presented reduced levels of apoptosis and were resistant to PIs [160].

Conversely, the translocations t(11;14) and t(6;14) do not confer a bad prognosis, occurring in 15% or 3% of MM patients, respectively [155,165]. The t(11;14) upregulates CCND1, resulting in better response to bortezomib therapy [177]. Nonetheless, some studies claim that CCND1 upregulation may be related to bad prognosis and MM progression, as the CCND1 amplification is associated with increased multidrug resistance 1 (MDR1) gene expression and chemoresistance [178]. The translocation t(6;14) upregulates CCND3 expression which, similarly to CCND1, regulates the cell cycle [155].

Secondary cytogenetic events frequently consist of deletions. Deletion of the short arm of chromosome 17 (17p13.1) confers a dismal prognosis in MM, associated with advanced stages of MM and drug resistance, essentially due to the loss of the p53 tumor suppressor gene (TP53) and consequent dysregulated control of cell cycle and apoptosis [165,179,180]. So far, all treatments including autologous stem cell transplantation [155], lenalidomide [181], bortezomib [122] or thalidomide-based are not effective in patients with this deletion [182]. The same occurs in relapsed or refractory patients [183], suggesting that del17p negatively influences all of the MM history [165,179,180].

Deletion of chromosome 13(q14q21), identified in 43% of the patients, is associated with an intermediate prognosis, mainly because of the simultaneous association with the t(4;14) and t(14;16) translocations, in 85% and 92% of patients, respectively [184]. Nonetheless, patients with the 13q deletion may benefit from bortezomib, having similar response to bortezomib when compared to those without this deletion [185].

Abnormalities in chromosome 1, in particular deletion of the 1p21 region and gain of the chromosome 1q arm, have been associated with shorter survival in MM [165]. Target genes implicated in deletion of 1p are FAM46C and CDKN2C, associated with reduced remission and overall survival in patients treated with ASCT [186,187]. Gains of 1q also affect the outcome of patients that underwent ASCT [123] or bortezomib-based therapy, confering bortezomib resistance. Nonetheless, IMIDs appear not to be affected by abnormalities in chromosome 1 [188].

Loss of the chromosomal region 8p21 is related to bad outcome of MM patients. The deletion of 8p21 is associated with a poor response to bortezomib and patients with this deletion showed less than 50% response whereas patients that carried normal 8p21 achieved 90% of response [124]. The 8p21 deletion confers resistance to bortezomib by upregulation of the decoy TNF-related apoptosis-inducing ligand (TRAIL) receptor, causing resistance of the abnormal PC to Apo2L/TRAIL mediated apoptosis. The use of immunomodulators, such as lenalidomide, could abrogate this resistance [189].

Overexpression of the MYC oncogene at the 8q24 chromosome occurs at a later stage of the MM pathogenesis. MYC abnormalities together with its co-localization with super-enhancers [190], are associated with disease aggressiveness, and to melphalan and bortezomib resistance [125,156].

Other abnormalities may be found in latter and aggressive stages of MM, mainly gene mutations involving gain of function of oncogenes such as NRAS, KRAS, BRAF and CCND1, loss of function of tumor suppressors like p53, RB1, DIS3, CDKN2A and CDKN2C, and mutations in the NF-κB and STAT3 pathways. These mutations play a major role in oncogenesis, promoting tumor progression and drug resistance [16,191,192].

Resistance due to mutations in the drug-targets are also observed in MM, mainly related to alterations in proteasome conformation in the case of PIs, or cereblon activity in the case of immumodulators [149,150,151]. Several mutations in proteasome subunits conferring resistance to bortezomib have been identified, resulting in overexpression of the proteasome subunit, or changes of the spatial arrangement in the proteasome inhibitor binding pocket, resulting in different degrees of resistance [150]. The most common mutations are in the proteasome subunit beta 5 (PSMB5) gene which encodes for the B5 subunit of the proteasome [149]. This subunit is responsible for the chymotrypsin-like proteasome activity and is the main target for bortezomib and carfilzomib. The first mechanisms described as being associated with resistance to IMIDs were mutations in cereblon (the common primary target for IMIDs), leading to its downregulation [151]. High expression of cereblon is associated with a good response to thalidomide, lenalidomide and pomalidomide, whereas low expression of cereblon is associated with weak or no response to IMIDS [70,72]. Moreover, some mutations were identified only in refractory and relapsed disease, probably due to clonal selection during long term use of IMIDs [151]. Other mutations related to cereblon function were also identified, leading to low levels of the cereblon binding protein IKZF1 and high levels of another cereblon binding protein, the karyopherin subunit alfa2 (KPNA2), which was correlated with lack of response to pomalidomide [70]. In fact, a study revealed the all cereblon-mutated patients and 90% of the cereblon pathway-mutated ones didn’t response to IMIDs based treatment [151].

### 3.2. Epigenetic Alterations and MicroRNAs Responsible for Drug Resistance in MM

Several epigenetic mechanisms were also linked with MM (Figure 3b): (i) hypomethylation of DNA and hypermethylation of tumor suppressor genes, leading to abnormal expression of important regulatory genes [20]; (ii) histone modifications promoting cell survival and cell cycle progression [16,166]; and (iii) abnormal miRs expression disturbing several pathways involved in MM pathogenesis [193,194].

In MM, hypomethylation is associated to disease progression, poor prognosis and drug resistance, by upregulation of the ABCG2 gene and, consequently, increased drug efflux [195]. On the other hand, hypermethylation of tumor suppressor genes was also shown to interfere on cell cycle, DNA repair, apoptosis and signaling pathways regulation [161]. 

Additionally, several miRs targeting important genes have been found dysregulated in MM, such as miR-21, miR-106b-25 cluster, miR-181a and b, miR-32 and miR-17-92 cluster [157,193,194,196]. For instance, miR-106b-25 cluster, miR-181a, miR-181b and miR-32 were found to be upregulated in MM, favoring oncogenesis due to their capacity to downregulate the p53 tumor suppressor by controlling the expression of PCAF, a histone acetyltransferase involved in transcription control of TP53 [197]. Conversely, miR-15a and miR-16 were found to be downregulated in MM cells when compared with normal PCs. In addition, miR-15a/miR-16, may have a protective function as tumor suppressor miRs by inhibiting the NF-κB pathway or decreasing VEGF secretion and, consequently, regulating tumor cell proliferation and suppressing angiogenesis [198].

Although some miRs were identified as being dysregulated in MM, few miRs were identified as having a role in drug resistance. The most interesting studies associated miR-21 upregulation with the development of dexamethasone and doxorubicin resistance in MM cells. Moreover, miR-21 was found upregulated in myeloma cells when bound to BMSCs, suggesting that the microenvironment is a key player in cell adhesion mediated drug resistance [199]. These findings support previous reports that observed miR-21 upregulated in the presence of IL-6 and via the activation of the STAT3 pathway [200]. On the other hand, miR-21 by decreasing PTEN, which is one of its tumor suppressor’s targets, upregulates the activity of two signaling pathways, the phosphatidylinositol 3-kinase/ protein kinase B (PI3K/AKT) and the mitogen-activated protein kinase/ extracellular signal-regulated kinase (MAPK/ERK) pathways, contributing to increased cell survival and drug resistance [201]. An additional study compared miRNAs expression between MM resistant cell lines and their parental sensitive counterparts, verifying that miR-21 was usually upregulated in melphalan-resistant MM cells [157,202]. Another interesting microRNA found dysregulated in MM patients is miR-15a, which usually acts as a tumor suppressor. However, in MM cells co-cultured with BMSCs (responsible for IL-6 release), miR-15a was downregulated by IL-6, causing enhanced protection from apoptosis induced by bortezomib and melphalan. Conversely, when cells were sensitive to those drugs, the expression levels of miR-15a were restored and MM cell death was achieved [126]. Another miRNA with the same tumor suppressor role is miR-29b which, when overexpressed in malignant MM cells, induces apoptosis by inhibiting the anti-apoptotic gene myeloid cell factor 1 (Mcl-1) [203]. Furthermore, overexpression of miR-29b sensitized MM cells to bortezomib [204]. Finally, miR-33b was found to be inhibited in MM cells, and its upregulation resulted in enhanced MM cellular apoptosis and sensitivity to ixazomib treatment [127]. In conclusion, microRNAs dysregulation contributes to the development of a MM drug resistant phenotype. As knowledge is being gained about their functional roles and involved pathways, the possibility of using miRs as targets for therapeutic strategies is becoming a promising approach for overcoming drug resistance in MM [157,196].

### 3.3. Abnormal Drug Transport

In cancer, the most frequent cause of drug resistance is abnormal drug transport, resulting in decreased intracellular drug levels (Figure 3c). This often occurs due to the overexpression of the MDR1 gene, that codes for the P-glycoprotein (P-gp, also known as MDR1 or ABCB1) drug efflux pump [205,206]. Indeed, the MDR1 gene and P-gp protein were previously found overexpressed in MM resistant cells [207]. Additionally, enhanced P-gp levels were found in MM patients after treatment with vincristine and doxorubicin, predicting MDR and cancer relapse. Indeed, relapsed patients presented significantly increased P-gp levels when compared with non-treated MM patients having low levels of P-gp expression [158,208,209]. Other drugs used in MM have been described as being P-gp substrates, namely dexamethasone, melphalan [117], lenalidomide [210], and carfilzomib [128,129]. After treatment with all of these drugs, except for melphalan, MM cells presented P-gp overexpression, mediating drug efflux and reducing therapeutic effects, thus contributing to the development of drug resistant cells [117,128,129]. Interestingly, thalidomide and bortezomib were described as weak P-gp substrates [117]. Nevertheless, some reports indicate that P-gp affects bortezomib activity, by decreasing both its function and expression [211]. 

In addition, breast cancer resistance protein (BCRP, also known as ABCG2) increased after treatment of MM cell lines with doxorubicin [158]. Nevertheless, low levels of another drug transporter mRNA, the MDR-associated protein 1 (MRP1, also known as ABCC1) mRNA, were detected in MM cells [208].

### 3.4. Escape from Apoptosis, Autophagy Activation and Dysregulated Intracellular Signaling Pathways

Other well-known mechanism of drug resistance found in MM patients is protection from drug-induced apoptosis (Figure 3d). Apoptosis is a programmed cell death process mediated by proteins and initiated by major signaling pathways, such as the NFκB, PI3K/AKT and the proteasome pathway [212]. The MAPK/ERK and the Janus kinase/signal transducer and activator of transcription 3 (JAK/STAT3) pathways, triggered by IL-6, have a crucial role in the induction of MM cellular apoptosis [213]. MAPK/ERK and PI3/AKT pathways can also be activated by other factors, such as, VEGF, fibroblast growth factor (FGF), stromal cell-derived factor 1α (SDF1α) [214] or insulin-like growth factor 1 (IGF-1) [215,216]. The Apo2L/TRAIL induces apoptosis of MM cell lines and human cells that developed resistance to dexamethasone, doxorubicin, melphalan and mitoxantrone [217]. In addition, its activation reverted bortezomib-resistance in MM cells by increasing apoptosis [218].

Mcl-1, a pro-survival protein, is associated with MM cell survival. Its inhibition rapidly induced apoptosis in MM cells and its overexpression contributed to relapse and disease severity across all stages [130,131,132]. Mcl-1 levels were found enhanced in MM cell lines in response to IL-6, following activation of the JAK/STAT3 pathway, and also in cell lines and primary cells in the presence of VEGF [219,220,221].

In some MM cells lines and primary cells a correlation was found between the MM phenotype and increased expression of Bcl-2 together with decreased Bax expression [222]. High levels of the Bcl-XL protein contributed to apoptosis inhibition through activation of the JAK/STAT3 pathway by IL-6. Indeed, Bcl-XL expression is also associated with MM drug resistance, having been found at higher levels in relapsed patients when compared to newly diagnosed ones [216].

NFκB, a family of five transcription factors, is commonly known for its anti-apoptotic effects contributing to malignant cells survival. NFκB is essential in MM pathogenesis and has been found to be constitutively active in myeloma cell lines and patient’s samples [133,223]. Moreover, it was found that drug sensitive MM cells display lower NFκB activity when compared with drug resistant ones, and that NFκB levels were higher in MM cells obtained from relapsed patients [52]. Thus, the NFκB blockage has been tested in a number of experiments by using arsenic trioxide, bortezomib or IκB kinase inhibitors, inducing apoptosis of myeloma cell lines [133].

MM cells are highly dependent on the unfolded protein response (UPR) pathway to restore homeostasis, since they have excessive levels of misfolded or unfolded proteins present within the endoplasmic reticulum (ER). The UPR is activated to decrease ER stress, leading to the inhibition of protein synthesis and increasing the transcription of heat shock protein (HSP) folding chaperones [134,224]. The remaining misfolded proteins present within the ER are then targeted for degradation by proteasome and autophagy [224,225,226]. To regulate the UPR pathway, different transcription factors enter the nucleus and activate UPR target genes [226]. This dependence on the UPR system and expression of UPR genes, makes MM cells more sensitive to PIs. For instance, bortezomib has a potent impact in MM cells since, by inhibiting the proteasome activity, causes the accumulation of misfolded proteins within the ER, which is fatal for the malignant cells that, consequently, undergo apoptosis [225]. Nevertheless, some patients develop bortezomib resistance. A correlation between the levels of a UPR key transcription factor, XBP1, and response to bortezomib was already found. Higher levels of XBP1 correlated with higher sensitivity to bortezomib [135]. Moreover, in vitro studies showed that reduced ER size as well as decreased ATF6 expression, a regulator of UPR and activator of the XBP1, are also correlated with bortezomib resistance. Taken together, these results suggest that decreased UPR activity may predict bortezomib resistance, but further studies are needed to establish this correlation in the clinical setting [134].

In MM cells, the induction of autophagy is not only necessary to collaborate with the UPR signaling but is also an important survival mechanism that cells use to degrade misfolded proteins and to survive. So, in MM, autophagy is associated with drug resistance. A role for autophagy in bortezomib resistance was observed when autophagy-inducer activating transcription factor 4 (ATF4) was found upregulated following treatment of different cancer cell lines with a proteasome inhibitor [136]. For that reason, strategies to target autophagy have been studied. Some approaches attempt to inhibit autophagy in order to induce apoptosis following drug treatment. In phase I and phase II clinical trials, the combination of autophagy inhibitors with bortezomib showed promising results for the treatment of relapsed or refractory patients [227,228]. In addition, the combination of carfilzomib with autophagy inhibitors potentiated apoptosis both in vitro and in vivo [229,230].

The heat shock proteins HSP70 and HSP90, players in chaperone-mediated autophagy (CMA), were associated with a variety of MM survival pathways [137]. HSP90 stabilizes proteins implicated in antiapoptotic signals such as AKT, STAT3 and IL-6 receptors [138]. Thus, HSP90 inhibition disturbs PI3/AKT, JAK/STAT3, MAPK/ERK and NF-κβ signaling pathways [138,139]. On the other hand, HSP90 has been previously described as a target of the JAK/STAT3, MAPK/ERK and also, via HSP70 expression, of the PI3K/AKT signaling pathway [137,140]. Inhibitors of HSP90 have been developed and tested in combination with other drugs for consequent apoptosis activation, such as bortezomib in vitro and in an orthotopic in vivo model [138,141,142] or inhibitors of the AKT pathway in vitro [140,231].

### 3.5. Persistence of Cancer Stem Cells

The persistence of cancer stem cells (CSCs, also called tumor initiating cells) within the heterogeneous tumor niche may contribute to justify the high rates of relapsed and refractory MM patients (Figure 3e). CSCs have been suggested as the main cells responsible for drug resistance development, due to their potential for self-renewal, differentiation capacity and ability to remain quiescent, slower cell cycle kinetics, enhanced capabilities such as DNA damage repair machinery, resistance to cell death mechanisms, overexpression of MDR efflux pumps, evasion from the immune response, adaptation to tumor microenvironment (TME) and greater cellular plasticity [115,232]. There are several studies with evidence for the presence of CSCs in MM, but proper definition and characterization of these cells always lacked consensus [233,234,235]. 

Some studies suggested that MM stem cells are CD138^−^ B cells. Indeed, human MM cell lines were shown to have small CD138^−^ subpopulations with greater clonogenic potential in vitro than the corresponding CD138^+^ cells. The clonogenic and resistant cells (CD138^−^ MM cells) displayed some stem cell properties, such as enhanced levels of ALDH activity. CD138^−^ cells from MM patients were also clonogenic both in vitro and in nonobese diabetic/severe combined immunodeficiency (NOD/SCID) mice, whereas CD138^+^ cells were not. It was assumed that MM CSCs were developed from populations of clonotypic B-cells. The CD138^−^ PCs phenotype was characterized by the surface markers usually found on normal B cells: CD19, CD20 and CD27 [234,235]. These clonogenic CD138^−^ MM precursors were relatively resistant to dexamethasone, bortezomib and lenalidomide. However, those drugs were able to inhibit CD138+ PCs growth [234,235,236]. Nonetheless, another study demonstrated that clonotypic CD138^+^ PCs also have some properties of CSCs such as self-renewal, tumour-initiating potential and drug resistance [237,238]. A more recent study with gene expression profiling of putative CSCs and the main population of MM cells derived from 11 MM patients, identified CD24+ MM cells as being capable of maintaining CSC features of self-renewal and drug resistance [239]. 

The pathways typically activated in CSCs, namely Hedgehog, Wnt and Notch, are also found highly activated in MM CSCs, being essential for their development and maintenance as well as for mediating the activation of drug efflux pumps, such as the ABCG2 [118,143]. These signaling pathways are triggered by autocrine signals and cytokines released from the BM microenvironment [118]. In MM, the Hedgehog pathway regulates stem cell fate and is particularly active in a minor fraction of cells, the CD138- PCs. The inhibition of this pathway results in the differentiation of these clonogenic cells [240]. The stimulation of the Wnt pathway and consequent accumulation of β-catenin maintains the proliferative capacity of MM cells, as it happens in hematopoietic stem cells and CSCs [152]. The Notch pathway is known to contribute to MM survival and proliferation. However, even though it was found highly expressed on clonotypic B cells, the role of Notch on these cells remains unclear [241,242]. MM CSCs also present enhanced expression of the retinoic acid receptor a2 (RARa2), causing drug resistance [144].

### 3.6. Tumor Microenvironment

The interaction between MM cells and a dysregulated BM microenvironment also contributes to chemotherapy resistance, known as environment-mediated drug resistance [115,119]. This type of acquired resistance can be divided into two categories: soluble factor-mediated drug resistance (SFM-DR) and cell adhesion mediated drug resistance (CAM-DR) (Figure 3f). The first comprises all the cytokines and growth factors secreted into the bone marrow milieu and the second includes the adhesion of myeloma cells to stromal cells, such as fibroblast and other BMSCs or to extracellular matrix (ECM) components, such as fibronectin [115,119,243]. The BM microenvironment also comprises several cell components, hematopoietic stem cells (HSCs), immune cells, erythrocytes, progenitor and precursor cells, bone marrow endothelial cells (BMECs), osteoclasts and osteoblasts [120]. 

The major soluble factors released are: IL-6, IGF-1, VEGF, B-cell activating factor (BAFF), FGF, SDF1α, and TNF-α. All these factors are secreted reciprocally between MM cells and BMSCs, representing an important mechanism to support myeloma cells survival [120,166]. This crosstalk network between BMSCs and MM cells also triggers signaling pathways activation, particularly the IL-6/JAK/STAT3 pathway [244]. IL-6 overexpression is possibly involved in resistance to several chemotherapeutic drugs, including bortezomib [126,146]. For that reason, several inhibitors of the IL-6/JAK/STAT3 pathway have been tested in order to prevent MM proliferation and induce apoptosis. These inhibitors have shown, both in vitro and in mouse xenograft models, optimistic results when tested alone or in combination with conventional therapies, such as dexamethasone [245,246], melphalan [247,248], bortezomib [248,249] and lenalidomide [250].

Constitutive activation of the pro-survival pathway NFκB controls IL-6 secretion, contributing to MM cells adhesion to BMSCs [251]. Bortezomib and thalidomide are capable of stimulating apoptosis by decreasing cytokines release into the BM milieu and overcoming drug resistance as well as the growth advantage of myeloma cells [252,253]. IGF-1 produced by the MM cells and present in the BM environment, promotes proliferation and drug resistance through activation of the MAPK and PI3/AKT pathways [254]. Increased VEGF secretion also enhances adhesion of MM cells and BMSCs. Additionally, this adhesion between cells increases IL-6 secretion by BMSCs, which in turn can increase the levels of VEGF that are secreted by myeloma cells (and vice-versa) [255]. Enhanced VEGF levels in the microenvironment promote angiogenesis and contribute to MM cell proliferation and migration. On the other hand, IL-6, VEGF and IGF-1, produced by BMECs, stimulate myeloma cells growth [120,166,255]. Several VEGF inhibitors have been developed in order to circumvent MM proliferation, survival and related drug resistance. These inhibitors increased MM cellular apoptosis in the presence of BMSCs, by decreasing IL-6 and VEGF secretion [256]. Some inhibitors, and antiangiogenic agents such as lenalidomide, showed synergistic effects with melphalan and bortezomib [257]. Another cytokine, the TNF-α, regulates adhesion between MM cells and BMSCs by increasing the levels of distinct cellular adhesion molecules (CAMs). The CAMs located at the cell surface of MM cells are the lymphocyte function-associated antigen 1 (LFA1) and very late antigen 4 (VLA4), while the CAMs at the cell surface of BMSCs are the intercellular adhesion molecule 1 (ICAM1) and vascular cell adhesion molecule 1 (VCAM1) [120,166]. In MM cell lines, the adhesion of myeloma cells, via VLA4, to the ECM component fibronectin prevented apoptosis and contributed to doxorubicin and melphalan resistance [159]. Concerning SDF-1/CXCL12, it is constitutively expressed and released by BMSCs and fibroblasts, while its receptor C-X-C chemokine receptor type 4 (CXCR4) is found in MM cells. The activation of the SDF1/CXCR4 axis promotes trans-endothelial migration, bone marrow homing, migration and adhesion of MM cells. The CXCR4 expression is correlated with bortezomib resistance in cell lines and the use of CXCR4 inhibitors may enhance the sensitivity of MM cells by disrupting their adhesion to the BMSCs [147]. Another potential cause for drug resistance in MM is activation of the myristoylated alanine-rich c-kinase substrate (MARCKS) membrane protein, that has an important role in cell adhesion and metastatic spread. MARCKS is activated by phosphorylation of protein kinases C (PKCs), being associated with bortezomib resistance. Inhibition of MARSCKS phosphorylation enhanced, in vitro, the sensitivity of resistant MM cells to bortezomib [148].

More recently, extracellular vesicles (EVs) have been associated with MM growth, progression and drug resistance [145,258,259,260]. EVs are secreted by different cell types and transport important molecules in their cargo, mediating intercellular communication [261,262,263,264]. In MM, EVs may be secreted by MM cells or by other cells in the tumor microenvironment. For instance, small EVs derived from MM bone marrow mesenchymal stromal cells (BM-MSCs) had increased levels of oncogenic proteins, cytokines and adhesion molecules when compared with small EVs released by normal BM-MSCs. In addition, interesting results showed that EVs released from primary cultures of BM-MSCs of relapsed or refractory MM patients have downregulated levels of miR-15a, a tumor suppressor miR, when compared with EVs from primary BM-MSCs obtained from normal healthy subjects [145]. This suggests that EVs facilitate the communication between the MM cells and the BM microenvironment, further supporting a role for the tumor microenvironment in disease progression [145,258].

### 3.7. Other Specific Mechanisms for Immunotherapies with Antibodies

Some MM patients are non-responsive and therefore resistant to treatment with mAbs (Figure 3g). The mechanisms behind this lack of response and drug resistance are not fully understood [78]. Preclinical data suggests that the levels of CD38 expression seem to be associated with daratumumab-mediated ADCC and CDC [154]. In the clinical practice, pre-treatment CD38 levels were higher in patients who achieve at least a partial response, when compared to those who did not. Indeed, CD38 was highly expressed before treatment and significantly decreased over time, including by the stage of progressive disease in which patients expressed low levels of CD38 levels [153]. Upregulation of cell surface expression of the regulation complement-inhibitor proteins CD46, CD56 and CD59 were found to be associated with daratumumab resistance, by blocking antibody-induced CDC [153,154]. Soluble forms of CD38 and SLAM7 may affect the activity of daratumumab and elotuzumab, respectively, by the extracellular binding of the mAbs to target antigens thereby reducing the specific binding to PCs [78]. Another mechanism of resistance described is the development of anti-idiotype antibodies that neutralize the activity of the therapeutic mAbs before reaching their specific cellular targets [99].

## 4. Concluding Remarks

The introduction of new drugs and combined regimens improved the overall survival of MM patients in recent years. However, drug resistance is still a concern to the majority of patients and particularly for the ones that relapsed or became refractory to those novel therapies. Understanding the different mechanisms of drug resistance will allow the identification of new targets and the development of novel drugs to counteract this clinical problem. This review highlighted the major novel available therapeutics for MM and causes of drug resistance. Of note, MM tumor cells may acquire simultaneously various alterations responsible for drug resistance, being particularly relevant when occurring in CSCs. Most importantly, the alterations in MM cells do not justify all cases of drug resistance, and attention needs to be paid to alterations in the tumor microenvironment and to intercellular communication. 

It is imperative to be able to classify MM patients and to define at an early stage of the disease appropriate personalized therapeutic strategies. Thus, it will be necessary to fully understand the molecular mechanisms involved in drug resistance for different drugs, in order to identify new molecular targets and therapeutic tools to overcome this problem and prolong MM patients’ survival. 

## Figures and Tables

**Table 1:** Mechanisms of action and drug resistance of the most used classes of drugs in Multiple Myeloma.

Agents	Mechanism of Action	Type of Resistance	Mechanism of Resistance
Proteasome Inhibitors (bortezomib, carfilzomib and ixazomib)	Inhibition of proteasome activity; inhibition of NF-κB; induction of apoptosis by activating caspase-8 and caspase-9; upregulation NOXA; down-regulation of adhesion molecules [52,53,55,61].	Genetics and Genomics	Mutations of gene TP53; mutation of gene MAF, t14;16) and t(14;20); point mutations of the gene PSMB5 with overexpression of B5 subunit; upregulation of the proteasomal system; overexpression of the gene MYC; 1q21 gain; modification or loss of 8p21 [122,123,124,125].
		Epigenetics	downregulation of miR-15a; downregulation of mir-33b [126,127].
		Abnormal Drug Transport	Upregulation of P-gp (mainly for carfilzomib) [128,129].
		Escape from apoptosis, autophagy and dysregulated intracellular signaling pathways	Upregulation of pro-survival proteins (Mcl-1, Bcl-2, Bcl-XL); constitutive activation of the NF-κB pathway; Activation of the aggresome and autophagy pathway; Low levels of the UPR transcription factor XBP1 and autophagy-inducer activating transcription factor 4; increase in heat shock proteins (Grp78, Hsp90, Hsp70, Hsp8) [130,131,132,133,134,135,136,137,138,139,140,141,142].
		Persistence of Cancer Stem Cells	Stem cell-like phenotype with increased levels of multidrug transporters (ABCG2/BCRP) and ALDH1A1 enzymatic activity; Activation of Hedgehog, Wnt and Notch pathways; upregulation of BTK receptors and RARa2 [118,143,144].
		Microenvironment	Proliferation and cell survival signaling such as IL6/JAK/STAT3, MAPK, PI3/AKT, IGF-1; Increase production of VEGF leading to angiogenesis, cell proliferation and migration; Increase of pro-inflammatory TNF-α; Increase of cell adhesion molecules (LFA1, VLA4, ICAM1, VCAM1); Activation of SDF1/CXCR4 axis; Increase expression of MARCKS in adhesion and metastatic spread; EVs cargo and cell communication [126,145,146,147,148].
Immunomodulatory agents (thalidomide, lenalidomide, pomalidomide)	Interaction with BM microenvironment with down-regulation of adhesion molecules; targeting the cereblon and downstream targets; regulation of pro and anti-inflammatory cytokines; regulation of T cell and NK cells activity; anti-angiogenesis; induction of apoptosis by activating caspase 8 and 9 [64,65,66,67,68,69,70,71].	Reduced target expression	Mutations in cereblon and genes in the cereblon-pathway (IFKF1 and KPNA2); Mutations in Ras/Raf pathway (KRAS G12D and BRAF V600E) [70,149,150,151].
		Genetics and Genomics	Mutations in cereblon and genes in the cereblon-pathway (IFKF1 and KPNA2) [151].
		Persistence of Cancer Stem Cells	Stem cell-like phenotype with increased levels of multidrug transporters (ABCG2/BCRP) and ALDH1A1 enzymatic activity; Activation of Hedgehog, Wnt and Notch pathways; upregulation of BTK receptors and RARa2 [118,143,144].
		Microenvironment	Increase of cell adhesion molecules (CD44 thought the Wnt/B-catenin signaling) [152].
Monoclonal antibodies (daratumumab, elotuzumab, isatuximab)	Antibody-dependent cellular cytotoxicity; complement-dependent cytotoxicity; macrophage-mediated phagocytosis; FCyR-mediated crosslinking; modulation of target antigen enzymatic activity; regulation of Tregs and stimulation of T cell and NK activity [79,81,85,91,92,96].	Reduced target expression	Reduced expression of CD38 and SLAM7 [153].
		Complement resistance	Increased expression of CD46, CD56 and CD59 blocking anti-body-induced CDC [153,154].
		Microenvironment	Competition by the extracellular soluble forms of CD38 and SLAM7 [78].
		Neutralization	Anti-idiotype antibodies neutralizing the activity of the therapeutic monoclonal antibodies [99].
Histone deacetylase inhibitors (panobinostat, vorinostat)	Activation of tumour suppressor genes; synergetic activity with proteasomal and aggresomal protein degradation; upregulation p21 [101,102].	Escape from apoptosis, autophagy activation and dysregulated intracellular signaling pathways	Abnormal regulation of actin cytoskeleton and abnormal protein processing in endoplasmic reticulum (activation of PI3/AKT, MEK/ERK and FAK signaling pathway) [137,138,139,140]
Exportin 1 inhibitors (selinexor)	Nuclear retention and activation of tumour suppressor genes, inhibition of NF-κB; reduction of oncoprotein mRNAs translation [109,110].	-	-
Alkylating agents (melphalan, cyclophosphamide) and Anthracyclines (doxorubicin)	DNA damage; topoisomerase II inhibition	Genetics, Genomics and Epigenetics	Overexpression of the gene MYC; upregulation of miR-21; downregulation of miR-15a [126,155,156,157].
		Abnormal Drug Transport	Upregulation of P-gp; increased ABCG2 expression [117,158].
		Persistence of Cancer Stem Cells	Stem cell-like phenotype with increased levels of multidrug transporters (ABCG2/BCRP) and ALDH1A1 enzymatic activity; Activation of Hedgehog, Wnt and Notch pathways; upregulation of BTK receptors and RARa2 [117,118,144,152,158].
		Microenvironment	Increase of cell adhesion molecules (VLA4) [159].
Corticosteroids (dexamethasone, prednisolone, methylprednisolone)	Induction of apoptosis	Reduced target expression	Functional defect of the glucocorticoid receptor [114,115,116,117].
		Genetics, Genomics and Epigenetics	Overexpression of the gene MYC and FGFR3; epigenetic inactivation of RASD1 [114,115,116,117].
		Microenvironment	Increase secretion of pro-survival cytokines [114,115,116,117]
